# Ferroptosis of T cell in inflammation and tumour immunity

**DOI:** 10.1002/ctm2.70253

**Published:** 2025-03-05

**Authors:** Xueli Xia, Haisheng Wu, Yuxuan Chen, Huiyong Peng, Shengjun Wang

**Affiliations:** ^1^ Department of Laboratory Medicine Jiangsu Province Engineering Research Center for Precise Diagnosis and Treatment of Inflammatory Diseases Affiliated Hospital of Jiangsu University Zhenjiang China; ^2^ Department of Immunology Jiangsu University School of Medicine Zhenjiang China; ^3^ Qinghai Provincial Institute of Endemic Disease Prevention and Control Xining China; ^4^ Department of Laboratory Medicine Affiliated People's Hospital Jiangsu University Zhenjiang China

**Keywords:** CD4^+^ T cell, CD8^+^ T cell, ferroptosis, inflammation, tumour

## Abstract

Ferroptosis is an innovative concept defined as a distinct programmed cell death mode regulated by iron‐dependent lipid peroxidation accumulation. This process is governed by numerous energy metabolites such as fatty acids, amino acids and glucose, as well as iron homeostasis. In recent years, increasing studies have been devoted to the crucial effects of ferroptosis in immune cells during the pathogenesis of diseases such as infections, tumours and autoimmune disorders. This review summarises the latest advancements in T‐cell ferroptosis, addresses the key components and mechanism of ferroptosis in T cells during inflammatory conditions and tumour progression, and highlights the potential target for treating related diseases.

**Key points:**

- Ferroptosis‐related mechanisms significantly affect the biology of CD4^+^ T‐cell subsets and are further involved in inflammatory diseases.
- Crosstalk between CD8^+^ T cells and tumour cells induces ferroptosis in the tumour microenvironment.
- Glutathione peroxidase 4 loss promotes regulatory T‐cell ferroptosis to enhance anti‐tumour immunity.

## Graphical abstract

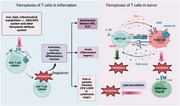

## 1 INTRODUCTION

Ferroptosis is a regulated cell death process related to iron homeostasis disruption, which features mitochondrial dysfunction and lipid peroxidation such as excessive iron‐mediated reactive oxygen species (ROS) generation.^1^ Ferroptosis has a unique morphology, biochemistry and genetic characteristics that diverge from necrosis, apoptosis and autophagy. Ferroptosis neither display the classical hallmarks of apoptosis nor does it result in the formation of autophagic vacuoles.^2^ Particularly, ferroptosis is manifested by mitochondria morphological changes, including shrunk mitochondria, elevated membrane density, and decreased or absent cristae, but maintains the early cell membrane integrity and unaltered nuclear morphology.^2^, ^3^ Recently, ferroptosis has also been indicated as a type of regulated necrosis characterised by plasma membrane rupture and release of cytoplasmic contents that often result in tissue damage and intense inflammatory responses.^4^, ^5^

**FIGURE 1:**
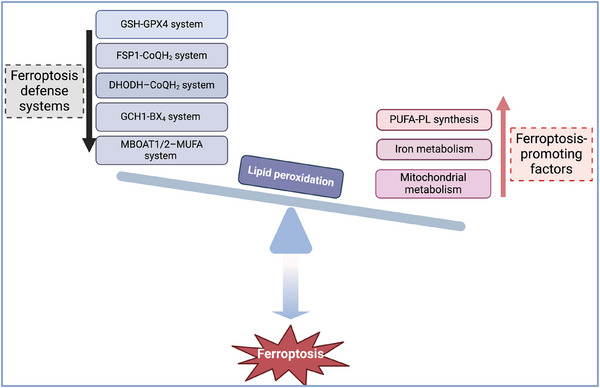
Imbalance between factors promoting ferroptosis and the defense system leads to ferroptosis. Ferroptosis‐promoting factors include polyunsaturated fatty acid‐containing phospholipids (PUFA‐PLs) synthesis, iron metabolism and mitochondrial metabolism. Conversely, ferroptosis defense systems involve the GSH‒GPX4 system, the FSP1‒CoQH_2_ system, the GCH1‒BH_4_ system, the DHODH‒CoQH_2_ system, and the MBOAT1/2‒MUFA system. When the activities of ferroptosis‐promoting factors surpass the detoxification capabilities provided by ferroptosis defense systems, unsaturated fatty acids on the cellular membrane undergo lipid peroxidation under the action of divalent iron or lipoxygenases, leading to the occurrence of ferroptosis. BH_4_, tetrahydrobiopterin; CoQH_2_, ubiquinol; DHODH, dihydroorotate dehydrogenase; FSP1, ferroptosis suppressor protein 1; GCH1, GTP cyclohydrolase 1; GPX4, glutathione peroxidase 4; GSH, glutathione; MBOAT1/2, membrane‐bound O‐acyltransferase domain containing 1/2; MUFA, monounsaturated fatty acid.

The initiation of ferroptosis signifies a subtle disruption in the equilibrium between factors that promote ferroptosis and the defense mechanisms (Figure 1). Currently, the cardinal mechanism‐regulated ferroptosis include polyunsaturated fatty acid‐containing phospholipid (PUFA‐PL) synthesis and lipid peroxides accumulation, iron metabolism and mitochondrial metabolism.^6^ The main defense systems against ferroptosis consist of the antioxidant system Xc^−^‐reduced glutathione (GSH)‐GSH peroxidase 4 (GPX4) system, the ferroptosis suppressor protein 1 (FSP1)‐ubiquinol (CoQH_2_) system, the GTP cyclohydrolase 1 (GCH1)‐tetrahydrobiopterin (BH_4_) system, the dihydroorotate dehydrogenase (DHODH)‐CoQH_2_ system, and the recently identified membrane‐bound O‐acyltransferase domain‐containing 1/2‐monounsaturated fatty acids (MBOAT1/2‐MUFA) system.^6^, ^7^, ^8^

Ferroptosis plays pivotal roles in immune cells such as T cells,^9^, ^10^ neutrophils^11^, ^12^ and macrophages.^13^, ^14^ It has also become the focus and hotspot of investigation into the underlying causes of inflammation, autoimmune conditions and tumourigenesis. The balance between T‐cell activation and elimination is fine tuned to prevent autoimmune diseases and ensure effective immune responses. Extensive evidence confirmed that distinct ferroptosis‐related metabolic signals, for instance, the mammalian target of rapamycin‐1 (mTOR) signalling, glutaminolysis and metabolic byproducts such as ROS affect the differentiation, function and viability of T cells.^15^, ^16^, ^17^ The generation of specific ROS types such as hydrogen peroxide and superoxide anion in T cells can be triggered by T‐cell receptor (TCR)‐mediated phagocyte‐type nicotinamide adenine dinucleotide phosphate (NADPH) oxidase activation.^18^, ^19^ On the other hand, excessive ROS accumulation disrupts cellular redox balance and oxidative defense to promote ferroptosis.^20^, ^21^ Ferroptosis disrupts the survival and expansion of effector T cells that exacerbate pathogen invasion and tumour progression.^22^ Moreover, ferroptosis directly inhibits the differentiation and stability of regulatory T cells (Tregs), which promotes autoimmune diseases but hampers tumour growth.^23^, ^24^ However, the ferroptosis of T cells has been studied only then just started. In this review, we depicted the core mechanisms underlying ferroptosis in T cells and comprehensively analysed the unique manifestations of immune imbalance among different T‐cell subsets during tumour progression and in other inflammatory conditions. We detailed the crucial roles of key ferroptosis regulators in T‐cell differentiation and function, as well as the impact of various energetic metabolic alterations in T cells on their abnormal differentiation and the occurrence of ferroptosis. Furthermore, we emphasised that metabolic enzymes and ferroptosis‐related agents hold the potential for modulating T‐cell‐mediated adaptive immune responses in tumours and inflammation.

## 2 REGULATORS OF FERROPTOSIS IN CD4^+^ T CELLS

**FIGURE 2:**
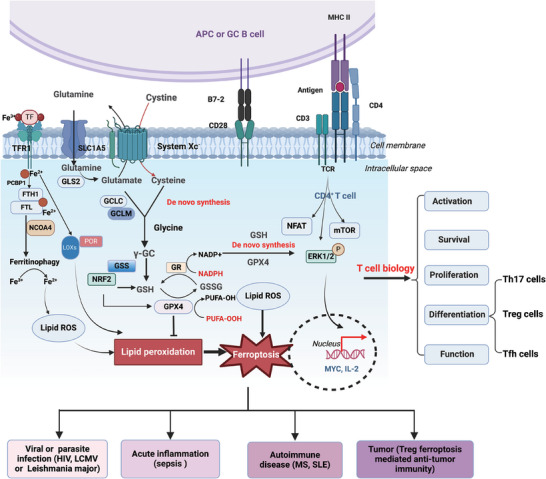
Ferroptosis process in CD4^+^ T‐cell biology. Iron metabolism, which involves the absorption, transport, distribution, storage, utilisation and excretion of iron, affects the ferroptosis sensitivity. Transferrin receptor 1 (TFR1)‐mediated iron transport and ferritin heavy chain 1 (FTH1)‐mediated iron storage regulate T‐cell activation and differentiation. System Xc^−^ functions as a cystine/glutamate antiporter that imports one molecule of cystine in exchange for one molecule of intracellular glutamate. In the process of de novo synthesis, γ‐GC ligase (GCL), which consists of a heterodimer of a catalytic subunit (GCLC) and a modifier subunit (GCLM), catalyses the first and rate‐limiting step to form the dipeptide γ‐GC from cysteine and glutamate. Glutathione (GSH) can be regenerated from oxidised glutathione disulphide (GSSG) by glutathione peroxidase 4 (GPX4), while GSSG is converted back to GSH via glutathione reductase (GR). The system Xc^−^‒GSH‒GPX4 axis affects T‐cell biology, such as activation, proliferation, survival, differentiation and function via ferroptosis and T‐cell receptor (TCR)‐mediated metabolic signalling pathways, which are further involved in infection, inflammation, autoimmune diseases and anti‐tumour responses. APC, antigen‐processing cell; ERK1/2, extracellular regulated protein kinase1/2; FTL, ferritin light chain; γ‐GC, γ‐glutamyl cysteine; GSS, glutathione synthase; HIV, human immunodeficiency virus; LCMV, lymphocytic choriomeningitis virus; LOX, lipoxygenase; MHC, major histocompatibility complex; MS, multiple sclerosis; mTOR, mammalian target of rapamycin; NFAT, nuclear factor of activated T cells; NRF2, NF‐E2‐related factor 2; POR, cytochrome P450 oxidoreductase; PUFA, polyunsaturated fatty acid; SLE, systemic lupus erythematosus.

Recently, ferroptosis has emerged as a vital contributor to the death of CD4^+^ T cells. The discussion on the ferroptosis mechanisms has been exhaustively elaborated in several outstanding reviews.^2^, ^21^ In this context, we briefly introduce the ferroptosis regulators and highlight their roles in CD4^+^ T cells. The mechanisms of ferroptosis in T cells and their effects on T‐cell biology and diseases are summarised in Figure 2 and Table 1.

**TABLE 1:** Regulators of ferroptosis in T cells and diseases.

Regulators	Function	Ferroptosis	T‐cell subsets affected	Diseases
ACSL4	Promote PUFA‐PLs synthesis	↑	T cells,^29^ CD8^+^ T cells^139^	Melanoma, colorectal cancer
FABP5	Lipid chaperone	↓	Tregs^131^	T‐cell lymphoma
TFR1	Iron importer	↑	T cells,^42^, ^43^, ^44^, ^45^, ^93^ Th1/Th2 cells,^50^ Th17 cells,^52^, ^111^, ^112^ Tregs^123^, ^124^	MS, SLE, IBD, HIV infection
FLVCR1	Haem transporter	↓	Naive CD4^+^ T cells and effector CD4^+^ T cells^41^	N/A
FTH1	Iron storage	↓	Tregs^24^, ^112^	SLE, MS, malaria, melanoma
PCBP1	Iron chaperone	↓	CD4^+^ T cells^52^	MS
GLS	Convert glutamine to glutamate	↑	CD4^+^ T cells,^100^, ^101^ Th1 cells,^100^ Th17 cells^100^, ^116^	SLE, chronic graft versus host disease, IBD
GCLC	Convert glutamine and cysteine to GSH	↓	Th17 cells^94^, ^115^ Tregs,^94^, ^114^ T cells,^95^ CD8^+^ T cells^158^	Melanoma, colorectal cancer, MS, IBD, LCMV infection, *C. rodentium* infection
GPX4	Degrade lipid peroxides	↓	CD8^+^ T cells,^22^, ^161^ T cells,^22^, ^83^, ^96^, ^97^, ^98^, ^99^ Tfh cells,^22^, ^104^ Tregs^23^	Melanoma, colorectal cancer, pancreatic cancer, LCMV infection, parasite infection, MS, liver transplantation
FSP1	Reduce CoQ_10_ or vitamin K to produce CoQH_2_	↓	CD8^+^ T cells^139^	Melanoma
NRF2	Regulator of the cellular antioxidant response	↓	Th17 cells,^107^, ^118^, ^119^ memory CD4^+^ T cells^107^	Colorectal cancer, MS, LCMV infection
CD36	Fatty acid translocase	↑	CD4^+^ T cells,^93^ CD8^+^ T cells^9^, ^140^	Melanoma, colorectal cancer
mTORC2	Promote NRF2‐mediated GPX4 activity persistence	↓	Memory CD4^+^ T cells^107^	LCMV infection
AMPK	Inhibit PUFA‐PLs synthesis	↓	CD8^+^ T cells,^76^, ^77^ Th17 cells,^77^ memory T cells,^78^ Tregs^134^	LCMV infection, colitis, melanoma, colorectal cancer, cervical cancer
VDAC	Control the output and entry of ions and metabolites	↑	Memory T cells^62^, ^63^	HIV infection
PGE2	Inflammatory mediator	↑	CD8^+^ T cells^155^	Melanoma

Abbreviations: ACSL4, acyl‐coenzyme A synthetase long‐chain family member 4; AMPK, adenosine‐monophosphate‐activated protein kinase; FABP5, fatty acid binding protein 5; FLVCR1, feline leukaemia virus subgroup C cellular receptor 1; FSP1, ferroptosis suppressor protein 1; FTH1, ferritin heavy chain 1; GCLC, glutamate‒cysteine ligase assembled from a catalytic subunit; GLS, glutaminase; GPX4, glutathione peroxidase 4; HIV, human immunodeficiency virus; IBD, inflammatory bowel disease; LCMV, lymphocytic choriomeningitis virus; MS, multiple sclerosis; mTOR, mammalian target of rapamycin‐1; N/A, not applicable; NRF2, nuclear factor erythroid 2‐related factor 2; PCBP1, poly(rC)‐binding protein 1; PGE2, prostaglandin E2; PUFA‐PL, polyunsaturated fatty acid‐containing phospholipid; SLE, systemic lupus erythematosus; TFR1, transferrin receptor 1; VDAC, voltage‐dependent anion channel.

### PUFA‐PLs synthesis

Free PUFAs serve as substrates for lipid peroxidation and could synthesise PUFA‐PLs with the assistance of labile iron and oxygen availability.^1^, ^25^ Acyl‐coenzyme A (CoA) synthetase long‐chain family member 4 (ACSL4) and lysophosphatidylcholine acyltransferase 3 (LPCAT3) are crucial regulators involved in PUFA‐PL synthesis.^26^, ^27^ Protein kinase C beta type isoform 2 (PKCβII) is a newly identified sensor to boost lipid peroxidation accumulation for the occurrence of ferroptosis via phosphorylation and activation of ACSL4.^28^ Morgan et al. employed mass spectrometry‐based targeted lipidomics to analyse the lipid deposition patterns in immune cells of both humans and mice (http://www.cellularlipidatlas.com).^29^ Interestingly, the PUFA‐PLs content was significantly abundant in lymphocytes, especially T cells, compared with myeloid cells. Consistently, lymphocytes with enriched PUFA‐PLs display high sensitivity to ferroptosis. Moreover, T cells with monounsaturated fatty acid (MUFA) supplementation concomitantly decrease the PUFA‐PLs content and protect T cells from ferroptosis. T cells with ACSL4 knockout display widespread alterations to PL acyl chain composition, decreased lipid peroxidation and further increased ferroptosis resistance.^29^ The expression of ACSL4 was enriched in multiple sclerosis (MS) patients and the experimental autoimmune encephalitis (EAE) mouse model, which induced neurons ferroptosis and inflammation.^30^ Ferroptotic neurons promote T‐cell activation and cytokine production in Th1 and Th17 cells via TCR, as well as induce T‐cell infiltration into the central nervous system. Lipid peroxidation products have been reported that may contain inhibitors of T‐cell response, increasing ROS intermediates and causing a shift towards an oxidative redox state, ultimately resulting in cell‐cycle arrest.^31^, ^32^ However, lipid peroxidation products can also significantly enhance CD4^+^ T‐cell proliferation and greatly benefit Th1 cell activation, further exacerbating autoimmune responses.^33^ Collectively, the PUFA‐PLs synthesis is a crucial mechanism for T‐cell ferroptosis.

### Iron metabolism

Iron metabolism, which involves the absorption, transport, distribution, storage, utilisation and excretion of iron, affects the ferroptosis sensitivity.^34^ Labile iron is transported into cells dependent on the transferrin receptor 1 (TFR1) and subsequently stored in ferritin containing ferritin heavy chain 1 (FTH1) and ferritin light chain (FTL).^34^ Ferritin can be degraded through a mechanism analogous to autophagy, termed ferritinophagy, causing labile iron release and ferroptosis initiation.^35^, ^36^ Significantly, nuclear receptor coactivator 4 (NCOA4) is regarded as an inducer of ferroptosis due to its importance in ferritinophagy.^37^, ^38^ Excessive labile iron facilitates ROS production through the Fenton reaction, thereby contributing to ferroptosis.^1^ Meanwhile, lipoxygenases and cytochrome P450 oxidoreductase (POR) both induce ferroptosis in an iron‐dependent manner.^25^, ^39^ Disruptions in iron metabolism, such as iron overload or deficiency, impact T‐cell activation and function.^40^ Deletion of feline leukaemia virus subgroup C cellular receptor 1 (FLVCR1) in naive CD4^+^ T cells shows characteristics with elevated intracellular iron storage, heightened spontaneous proliferation and hyperactive mitochondria.^41^ However, CD4^+^ effector T cells lacking FLVCR1 display an iron‐loaded phenotype and exhibit deficient proliferation and impaired mitochondria upon activation, driven by inadequate glycolysis and glutaminolysis, rendering them more susceptible to ferroptosis. Moreover, the surface TFR1 expression is upregulated via mTOR activation and interleukin (IL)‐2 signalling induces T‐cell development but is resistant to T‐cell anergy.^42^, ^43^, ^44^, ^45^ TFR1 mutation hinders iron endocytosis and induces T‐cell dysfunction, which causes a combined immunodeficiency.^46^ Inhibition of iron uptake with TFR1 blocking monoclonal antibody (mAb) induced non‐proliferating and altruistic T cells that through the bystander effect, released high amounts of interleukin (IL)‐2 for other immune cells.^47^ Iron supplementation decreases the surface expression of CD2 and CD4 ex vivo, while iron‐uptake inhibition affects Th1 cell differentiation more than Th2 cells.^48^, ^49^ Additionally, iron overload in CD4^+^ T cells can induce IL‐4 and IL‐10 generation while concurrently inhibiting interferon‐γ (IFN‐γ) production.^50^ TFR1‐mediated iron uptake also delivers an IFN‐γR2 internalisation signal in human T cells via inhibiting the IFN‐γ pathway cascade. In contrast, the application of an iron chelator has the opposite effects.^51^ Furthermore, dysfunctional intracellular iron has been observed under pathologic conditions. Intracellular iron shields RNA‐binding protein poly(rC)‐binding protein 1 (PCBP1) from proteolysis to augment the expression of granulocyte‐macrophage colony‐stimulating factor and IL‐2, thus enhancing pathogenic T cells activation in EAE.^52^ The elevated intracellular iron has also been discovered in self‐reactive T cells within systemic lupus erythematosus (SLE).^53^ Hence, iron metabolism is tightly related to T‐cell biology, but iron‐dependent ferroptosis in different T‐cell subsets during autoimmune diseases and tumour progression requires thorough exploration. Notably, the effects of NCOA4‐mediated ferritinophagy in T cells could be an investigative topic.

### Mitochondrial metabolism

Mitochondria serve as the location where various cellular energy metabolic events occur, playing a more prominent role in triggering ferroptosis. Cellular ROS were mainly produced through mitochondria glucose‐dependent bioenergetic processes and the electron transport chain transfer, which involved superoxide production and dismutase‐mediated H_2_O_2_ conversion, which can further connect with iron metabolism and promote PUFA‐PL peroxidation to drive ferroptosis.^54^ Excessive cytosolic iron catalyses the oxidised lipids via the Fenton reaction and stimulates lipoxygenases and POR activation, promoting hydroxyl radicals and oxidised PUFAs produced in mitochondria, which triggers the onset of ferroptosis.^2^, ^55^ ROS production is crucial for IL‐2 and IL‐4 generation in T‐cell activation and plays essential roles in T‐cell differentiation.^56^, ^57^ ROS such as H_2_O_2_ promotes the feedback loop between signal transducer and activator of transcription 6 (STAT6) activation and IL‐4 production in Th2 cells.^58^ ROS upregulated by glycolysis activates transforming growth factor‐β (TGF‐β) to favor Th17 cell differentiation, thus aggravating autoimmune disorders in colitis and EAE.^59^ Voltage‐dependent anion channel (VDAC) in the mitochondrial outer membrane controls the output and entry of ions and metabolites, which is required for mitochondria homeostasis and ROS production. It has also been demonstrated to be involved in Erastin‐induced ferroptosis.^60^, ^61^ The upregulated expression of VDAC was displayed in human immunodeficiency virus (HIV)‐1 infected CD4^+^ T cells and apoptotic memory T cells.^62^, ^63^, ^64^ In contrast, VDAC inhibitor 4,40‐diisothiocynostilbene‐2,20‐disulphonic acid could block the H_2_O_2_‐induced apoptosis in memory T cells. Moreover, the mitochondria are a prerequisite for the tricarboxylic acid (TCA) cycle, oxidative phosphorylation (OXPHOS) and glutaminolysis, which are tightly correlated with ferroptosis.^65^, ^66^, ^67^ Gao et al. further manifested that the TCA cycle only facilitates cysteine consumption‐mediated ferroptosis but has no effects on ferroptosis caused by GPX4 suppression in that the existence of glutaminolysis in mitochondria.^68^ The products of the TCA cycle such as alpha‐ketoglutarate, succinic acid and fumaric acid, effectively induce cysteine depletion‐mediated ferroptosis via lipid ROS generation and accumulation while blocking the activity of fumarate hydratase in the TCA cycle, which has anti‐ferroptosis effects.^68^, ^69^ Typically, the dynamic coordination of glucose metabolism pathways OXPHOS and glycolysis deeply influences the cellular ROS levels and could regulate ferroptosis susceptibility.^70^, ^71^ Interestingly, the dynamic glycolysis‐OXPHOS equilibrium is fundamental to the activation, development, and function of T‐cell subsets to meet their specific energy and biosynthetic requirements.^72^, ^73^ T effector cells prefer to increase glycolysis activity to differentiate into Th1, Th2 and Th17 phenotypes, while a shift from glycolysis to OXPHOS occurs during Treg differentiation.^74^ Under energy stress conditions such as glucose deprivation or ATP depletion, the activation of adenosine‐monophosphate‐activated protein kinase (AMPK) regulated by liver kinase B1 (LKB1) phosphorylates and inactivates acetyl‐CoA carboxylase (ACC), thereby inhibiting PUFA‐PLs synthesis and enhancing resistance to ferroptosis.^66^, ^75^ Meanwhile, the LKB1‒AMPK axis also plays a great role in T‐cell metabolic adaptation that regulates glutamine metabolism and mitochondrial bioenergetics to maintain T‐cell viability, differentiation and function.^76^, ^77^ Glucose limitation also activates AMPK and the subsequent SENP1‒Sirt3 signalling during T‐cell memory development.^78^ Above all, regulators involved in mitochondrial metabolism play multifold roles in T‐cell activation and differentiation, as well as in ferroptosis. Further studies should pay attention to the relationship between mitochondrial metabolism balance and T‐cell ferroptosis.

### GSH‒GPX4 system

The antioxidant GSH‒GPX4 system has been widely regarded as the crucial one of anti‐ferroptosis mechanisms to directly neutralise lipid peroxides. GPX4 can prevent intracellular lipid and cholesterol hydroperoxide product abundance due to its superior antioxidant capacity.^79^ The sources of intracellular GSH include the de novo synthesis process devoted by glutamate‒cysteine ligase (GCL) and glutathione synthase (GSS); the regenerated process from oxidised GSH (GSSG) is regulated by glutathione disulphide reductase.^80^, ^81^ During de novo synthesis, GCL is assembled from a catalytic subunit (GCLC) and a modifier subunit (GCLM), which induce γ‐glutamyl cysteine (γ‐GC) generation from cysteine and glutamate.^82^ When intracellular GSH depletion or exhaustion or decreased GPX4 activity, lipid peroxidation products cannot be detoxified by the GSH‒GPX4 axis, but accumulation due to iron metabolism and mitochondrial metabolism, therefore initiating ferroptosis.

GSH maintains T‐cell effector function by governing ROS elimination.^83^ Elevating intracellular GSH in T cells can enhance T‐cell activation and function, whereas GSH depletion significantly impairs T‐cell response to concanavalin A in vivo.^84^, ^85^ GSH homeostasis of CD4^+^ T cells is disturbed in HIV‐seropositive patients.^86^ Intracellular GSH levels maintained in T cells are associated with inhibiting cytokine‐dependent HIV replication ex vivo.^87^, ^88^, ^89^ Notably, T cells possessing elevated GSH levels are preferentially diminished in the early stages of HIV infection.^90^ HIV‐infected primary CD4^+^ T cells have been proven to exhibit typical ferroptotic characteristics along with iron homeostasis imbalance to further intensify HIV infection.^91^, ^92^ Moreover, the utilisation of anti‐retroviral therapy (ART) is unable to restore CD4^+^ T‐cell immunity but increases lipid peroxidation and disrupts the mitochondrial structure, which is associated with CD36 expression.^93^ The application of ferroptosis inhibitors was found to effectively alleviate inflammation and promote mitochondrial repair.^93^ Although GSH can be regenerated from GSSG, blocking this pathway does not seem to affect GSH levels or the fate of murine T cells. Conversely, suppressing the GSH de novo synthesis pathway reduces the GSH concentrations in T cells.^94^ GCLC loss severely impairs the activation, proliferation, as well as viability of T cells.^94^ GSH deficiency hinders mTOR activation and decreases the nuclear factor of activated T cells and Myc expression, which in turn alters the metabolic pathway essential to T‐cell activation and increases resistance to EAE.^6^, ^95^ In CD4 cre‐Gclc^fl/fl^ mice infected with lymphocytic choriomeningitis virus (LCMV), they also display diminished CD8^+^ T‐cell numbers and inflammatory cytokine production, resulting in high viral titers in CD4 cre‐Gclc^fl/fl^ mice. This underscores the crucial effects of GSH metabolism and ferroptosis in T cells for the prevention of autoimmune diseases but also highlights its importance in anti‐viral defense.

CD4^+^ T‐cell ferroptosis was caused by GPX4 downregulation in the acute inflammatory response.^10^ The specific knockout of GPX4 in T cells results in normal thymocyte development and maintains the CD4^+^ T‐cell homeostasis, especially Tregs.^22^ Meanwhile, the phenotypes of peripheral T cells show no significant variation, but the number of CD8^+^ T cells was decreased in mice with T‐cell‐GPX4 deletion.^22^ Additionally, GPX4 absence impairs the viability of proliferating T cells under non‐inflammatory conditions. Upon acute infection with LCMV or the protozoan parasite Leishmania major, T cells with GPX4 loss decrease the expansion ability and cannot eliminate the virus or parasite load. Yagoda et al. have proposed that the RAS‐mitogen‐activated protein kinase (MEK) signalling cascade was associated with ferroptosis.^96^ TCR activation induces extracellular regulated protein kinases (ERK1/2) phosphorylation via H_2_O_2_ production and glutamine to determine T‐cell fate and responses, while ERK1/2 pathway blockade prevents GPX4‐deficient T‐cell ferroptosis.^22^, ^83^, ^96^ The aberrant elevation of GPX4 expression observed in T cells of patients with MS has been found to be positively associated with increased immune cell recruitment and exacerbated inflammation.^97^ In contrast, GPX4 deletion in CD4^+^ T cells inhibits inflammatory T‐cell infiltration in EAE mice. Kang et al. have conclusively shown that GPX4 also acts as a crucial pyroptosis inhibitory factor in macrophages during severe inflammatory responses, which suggests the bifurcated effect of GPX4 in ferroptosis and pyroptosis and brings a new possible direction for the cross‐linking interaction between ferroptosis and pyroptosis in T‐cell function.^98^ Thus, GPX4 can be a promising therapeutic strategy for restraining inflammation and the CD4^+^ T‐cell ferroptosis process. Moreover, based on the importance of mTOR in ferroptosis and T‐cell biology, rapamycin, a mTOR‐specific inhibitor also as an autophagy inducer, combined with metronomic capecitabine could decrease GPX4 protein levels and further promote CD4^+^ T‐cell ferroptosis, which mediates immunosuppression after liver transplantation.^99^ This also implies the link between ferroptosis and autophagy in CD4^+^ T cells. Glutaminase (GLS) is a pivotal enzyme of glutamine metabolism, whose loss can inhibit initial T‐cell development.^100^ GLS2 has been proven to be an inducer of ferroptosis.^67^ GLS2 effectively diminishes ROS levels and lipid peroxidation accumulation by the GSH‐dependent antioxidant system and restores mitochondrial activity and activation‐associated IL‐2 expression in T cells.^101^ Moreover, the GLS2 expression in CD4^+^ T cells was decreased during the progression of SLE, which suggests the importance of glutamine metabolism and GSH metabolism in the pathogenicity of T cells. Thus, the GSH‒GPX4 axis is a vital mechanism to eliminate ROS and ferroptosis in T‐cell biology and pathogenicity, and it constitutes a significant intrinsic component of T‐cell maintenance. Notably, despite the absence of the system Xc^−^ cystine/glutamate transporter, GPX4‐deleted T cells are still capable of inducing ferroptosis. In the future, other defense mechanisms, such as FSP1 and GCH1‐regulated ferroptosis, should be investigated in T cells.

## 3 FERROPTOSIS IN T FOLLICULAR HELPER CELLS

T follicular helper (Tfh) cells represent a distinct subset of CD4^+^ T cells that assist B cells in enhancing germinal centre (GC) responses.^102^ As we mentioned above, intracellular iron is a key driver of ferroptosis. Iron accumulation mediated by the miR‐21/BDH2 axis and BCL6 gene demethylation via iron‐dependent epigenetic modulation differentiates CD4^+^ T cells towards Tfh cells, which further exacerbates antigen‐specific GC response during the pathogenesis of SLE.^103^ Yao et al. illustrated that Tfh cells have inferior survival rates than non‐Tfh effector T cells, exhibiting ferroptosis characteristics including morphological alteration of mitochondria and lipid peroxidation in mice immunised with SRBC or NP‐OVA or in mice with LCMV Armstrong infection.^104^ Consistent with the findings observed in mouse Tfh cells, human tonsillar Tfh cells and circulating counterparts alike demonstrate poor viability. Notably, the application of ferroptosis inhibitors has the potential to rescue Tfh cells from cell death. Moreover, these Tfh cells are found to be more sensitive to ferroptosis inducer RSL‐3 than non‐Tfh cells under anti‐CD3/CD28 stimulation.^104^ GPX4 is an intrinsic factor for Tfh responses. GPX4 deficiency in CD4^+^ T cells causes lipid ROS generation and shrunken mitochondria.^22^, ^104^ CD4 cre‐Gpx^fl/fl^ mice demonstrate a progressive and notable decrease in Tfh cell frequencies, accompanied by low expression of ICOS and an unchanged proliferation state. Consequently, these mice exhibited impaired GC formation and reduced B‐cell differentiation, leading to decreased high‐affinity antibody production following NP‐OVA immunisation. On the contrary, treatment with ferrostatin‐1 or dietary selenium supplementation can mitigate ferroptotic characteristics and increase ICOS expression, thereby boosting the Tfh response. Tfh cells interact with GC B cells via intensified TCR signal, leading to lipid peroxidation within Tfh cells and elevating their vulnerability to ferroptosis. Last but not least, GPX4‐deficient T cells exhibit enhanced expression of transcription factor GATA binding factor 3 (GATA3) for Th2 cells and an elevated frequency of Rorγt^+^ Th17 cells and Tregs but with comparable follicular regulatory T (Tfr) cells compared to wild‐type controls in mice with NP‐OVA immunisation, which suggests the preferential role of GPX4 for Tfh survival and function, as well as a distinct role of GPX4 in other CD4^+^ T‐cell subsets. The possible reason for the different susceptibility to ferroptosis may be that non‐Tfh cells receive transient and relatively weak TCR signals, which warrants further investigation in the future.^105^, ^106^

A recent study has revealed that the mTOR complex 2 (mTORC2)‒AKT‒GSK3β axis is imperative for the memory CD4^+^ T‐cell production and maintenance, especially Tfh and Th1 populations during LCMV infection.^107^ However, inactivation or ablation of mTORC2 cascade subsequently attenuates hexokinase 2 (HK2) bind to VDAC, decreases the nuclear translocation of nuclear factor erythroid 2‐related factor 2 (NRF2), a process vital for GPX4 activity persistence, contributing to an abnormal mitochondrial ROS accumulation and lipid peroxidation that triggers ferroptosis in memory CD4^+^ T cells following an acute LCMV infection.^107^ Additionally, it has been demonstrated that IL‐7‐CD127 engagement is crucial for the activation of the mTORC2‒AKT‒GSK3β axis and the homeostasis of memory CD4^+^ T cells, which also involves GPX4 peroxidase activity and ferroptosis.^108^, ^109^ Above all, given the high susceptibility of Tfh cells to ferroptosis and the importance of mTORC2 kinase in Tfh cell differentiation,^104^, ^110^ the comprehensive discovery of mTORC2‐mediated Tfh cell ferroptosis and the role of IL‐7 in ferroptosis become urgent issues in the future.

## 4 FERROPTOSIS IN TH17 CELLS

Although research on Th17 cell ferroptosis is limited, extensive attention has been paid to studying ferroptosis‐related molecules in the differentiation and functionality of Th17 cells. Iron metabolism exerts a critical effect on modulating the developmental trajectory of T cells in immune responses and diseases.^111^ Using anti‐TFR1 mAb to block the iron endocytosis compromised the differentiation of Th17 cells and mitigated EAE progression via inhibiting histone modifications of the *Il17* gene and reducing retinoid‐related orphan nuclear receptor γt (RORγt) recruitment. TFR1 blockade also decreased intracellular iron levels and mTORC1 signalling, subsequently suppressing Th1 and Th17 cell function while promoting the expansion of iTregs and enhancing IL‐10 secretion.^112^ In idiopathic inflammatory myopathies patients, iron can drive Th17 cell and Th1 cell polarisation via enhancing PFKFB4 through AKT‒mTOR signalling.^113^ Knockdown of GCLC preferentially drives Th17 cell differentiation but inhibits induced Treg (iTreg) differentiation ex vivo, based on the unique metabolic preferences of T‐cell subgroups.^94^ Interestingly, Kurniawan et al. revealed that iTregs contain more intracellular GSH than effector T cells.^114^ Therefore, we hypothesise that the two sources of GSH have different mechanisms of action in Th17 cells and Tregs. Bonetti et al. demonstrated that GSH loss via GCLC deletion reduces the IL‐22 production during *Citrobacter rodentium* (*C. rodentium*) infection.^115^ Th17 cells lacking GCLC display mitochondrial impairments and accumulation of ROS, which diminishes 4E‐BP1 phosphorylation and decreases IL‐22 synthesis, ultimately exacerbating intestinal inflammation. Additionally, GLS stimulates the differentiation and activity of Th17 cells by decreasing ROS levels, whereas it inhibits Th1 cells by modifying chromatin accessibility and gene expression patterns, ultimately enhancing IL‐2 and mTORC1 signalling pathways.^100^ In contrast, GLS1 inhibitor treatment can diminish RORγt expression and decrease the Th17 cell proportion.^100^ Peroxisome proliferator‐activated receptor (PPARγ) agonist treatment inhibits RORγt expression and abolishes Th17 responses through abrogating GLS1/GSH/ROS signalling.^116^ Interestingly, GPX4 has been reported to participate in suppressing Th17 responses, whereas GPX4 deletion enhances IL‐1β production, which in turn facilitates Th17 responses.^23^ Elevated frequency of RORγt^+^ Th17 cells was found in CD4 cre‐Gpx^fl/fl^ mice with NP‐OVA immunisation, which suggests the importance of GPX4 in Th17 cell differentiation.^104^ NRF2 increases the system Xc^−^ and GPX4 expression to influence cellular iron metabolism and redox homeostasis, thereby resisting ferroptosis.^117^ Enriched NRF2 promotes IL‐22 production through aryl hydrocarbon receptor pathway in Th17 cells.^118^ The p38‐regulated/activated protein kinase (PRAK)‒NRF2 axis has been recently confirmed to preserve intracellular redox homeostasis in Th17 cell differentiation and anti‐tumour functions.^119^ Hence, future research should delve deeper into the ferroptosis‐related mechanisms and pay attention to the intimate link between ferroptosis and Th17 cell pathogenicity.

## 5 FERROPTOSIS IN TREGS

Tregs play great roles in maintaining immune homeostasis, preventing autoimmunity and favouring tumour progression.^120^, ^121^ Iron homeostasis is essential to the function and survival of Tregs. Iron supplementation was associated with increased Tregs, further mitigating the immune responses in experimental cerebral malaria.^122^ Tregs with 3‐phosphoinositide‐dependent protein kinase 1 deletion disrupt the iron balance by modulating MEK‒ERK signalling and TFR1 expression, which causes glucose metabolism inhibition but excessive ROS generation, therefore increasing Treg apoptosis and ferroptosis.^123^ The expression of TFR1 was elevated in the autoreactive T cells during SLE progression.^112^ Iron deficiency reduces the levels of TFR1 and FTH1 in splenic CD4^+^ T cells, supporting Treg expansion and protecting Tregs from apoptosis via limiting ROS production in pristane‐induced lupus. Zhu et al. displayed that loss of TFR1 in Tregs reduced the differentiation of c‐Maf^+^ Tregs in the intestine and promoted lethal autoimmune disease.^124^ Furthermore, FTH1 is highly expressed in Tregs and supports forkhead box protein 3 (Foxp3) demethylation and Treg stability, ultimately affecting autoimmunity and anti‐tumour response.^24^ Collectively, iron balance is significantly involved in Tregs differentiation and suppressive capability.

A study reported that iTregs have less ROS production, more intracellular GSH levels, and higher mitochondrial function compared to Th0 cells.^114^ However, increased ROS generated from mitochondria in Tregs has been reported to promote Treg stability and suppressive function.^125^ Thus, the generation and scavenging of ROS in Tregs is a fine‐tuned process. GSH metabolism as a pivotal ROS scavenger is also involved in Treg function. GCLC loss in Tregs can elevate serine metabolism and ROS levels that impair the suppressive activity of Tregs, which further aggravates inflammation but hampers tumour growth.^114^ Furthermore, ROS levels were enriched in aged Tregs, while DCAF1 interacts with glutathione S‐transferase pi‐1 (GSTP1s) to buffer ROS in young Tregs, which restrains uncontrolled inflammation and immunological ageing.^126^

The abundance of intratumoural Tregs, possessing potent immunosuppressive capabilities, has a close association with unfavourable prognostic outcomes and poor survival rates among diverse cancer patients.^127^, ^128^, ^129^ Although Tregs deficient in GPX4 retain their homeostatic survival and expansion capabilities under steady‐state conditions, activated Tregs undergo significant lipid metabolism alteration and fatty acid oxidation, potentially leading to an increased generation of lipid peroxides.^22^, ^130^, ^131^ Fatty acid binding protein 5 (FABP5) is crucial for lipid transport and is involved in ferroptosis repression recently.^132^, ^133^ An earlier study has shown that the genetic or pharmacological downregulation of FABP5 in Tregs results in mitochondrial alterations such as decreased OXPHOS activity, compromised lipid metabolism, cristae structure loss and increased mtDNA‐induced cGAS‐STING activation, reinforcing the immunosuppressive capability of Tregs.^131^ Consistently, tumour‐infiltrating Tregs display similar mitochondrial changes.^131^ Recently, Pokhrel et al. have reported that energy sensor AMPK was markedly reduced in tumour‐infiltrating Tregs, which can enhance the PD‐1 expression of Tregs via the HMGCR/p38 MAPK/GSK3β signalling axis and further suppress tumour progression in an LKB1‐independent manner.^134^ These studies imply that the energy metabolisms associated with ferroptosis engage in maintaining the functionality of tumour‐infiltrating Tregs. Xu et al. have demonstrated that GPX4 is dispensable for maintaining Treg homeostasis.^23^ GPX4‐deficient Tregs exhibit elevated mitochondrial superoxide levels and lipid peroxidation and succumb to ferroptosis upon TCR stimulation and co‐stimulatory signals interaction. Furthermore, Tregs with GPX4 deletion promote the cell death of intratumoural Tregs while improving immune responses against tumours.^23^ Hence, GPX4 is an intrinsic critical determinant for preventing Treg ferroptosis and restraining immune activation in the progression of tumours. Recently, a research team has successfully developed a TFDD micelle that encapsulates ferric ion (Fe^3+^)‐coated doxorubicin (DOX), which can simultaneously trigger apoptosis and ferroptosis within the tumour microenvironment (TME) and facilitate the efficacy of immunotherapy.^135^ This apoptosis/ferroptosis inducer significantly impedes tumour growth and bolsters anti‐tumour response via effector T‐cell activation and simultaneously reduces the Treg‐to‐effector T‐cell ratio. This indicates that it could be a highly promising approach for advancing cancer immunotherapy.

## 6 FERROPTOSIS IN CD8^+^ T CELLS

**FIGURE 3:**
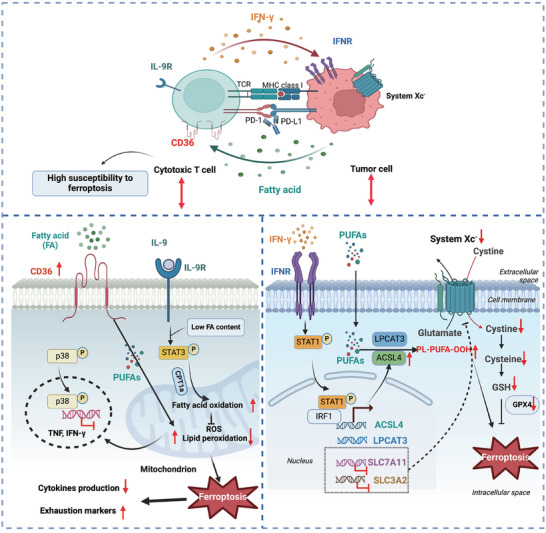
Ferroptosis crosstalk between CD8^+^ cytotoxic T cells and tumour cells. Ferroptosis has dual roles in anti‐tumour immunity. On the one hand, activated CD8^+^ T‐cell‐secreted interferon‐γ (IFN‐γ) interacts with IFN‐γ receptor (IFNR) on the tumour cells to activate STAT1/IRF1/ACSL4/LPCAT3 pathway, which catalyses the polyunsaturated fatty acids (PUFAs) into PUFA‐PL‐OOH and inhibit the system Xc^−^ (SLC7A11 and SLC3A2)‒GSH‒GPX4 axis, ultimately inducing tumour cells ferroptosis. On the other hand, elevated CD36 expression and cystine shortage in tumour‐infiltrating CD8^+^ T cells increase intracellular fatty acid uptake and lipid peroxidation overload, which triggers the exhaustion and ferroptosis of CD8^+^ T cells and impairs the anti‐tumour immunity. Moreover, a distinct subset of tumour‐infiltrating CD8^+^ T cells with interleukin‐9 (IL‐9) secretion have high fatty acid oxidation and resistance to ferroptosis, which can promote anti‐tumour immunity. ACSL4, acyl‐CoA synthetase long‐chain family member 4; CPT1a, carnitine palmitoyl transferase 1a; FA, fatty acid; GPX4, glutathione peroxidase 4; GSH, glutathione; IRF1, interferon regulatory factor 1; LPCAT3, lysophosphatidylcholine acyltransferase 3; MHC, major histocompatibility complex; PD‐1, programmed death 1; PD‐L1, PD1‐ligand; PUFA, polyunsaturated fatty acid; ROS, reactive oxygen species; SLC7A11, solute carrier family 7, member 11; SLC3A2, solute carrier family 3, member 2; STAT1/3, signal transducer and activator of transcription 1/3; TCR, T‐cell receptor; TNF, tumour necrosis factor.

The ferroptotic interaction between CD8^+^ cytotoxic T cells and tumour cells was reported in tumour immunity and concluded in Figure 3. Previous studies have manifested that tumour ferroptosis was elicited, and the anti‐tumour activity was reinforced by CD8^+^ T‐cell activation.^136^, ^137^, ^138^ On the other hand, CD8^+^ T cells within the intricate TME settings also display remarkable ferroptosis characteristics induced by diverse metabolites.^139^, ^140^ Therefore, we summarised CD8^+^ T‐cell‐induced tumour ferroptosis and CD8^+^ T‐cell ferroptosis, respectively.

### 6.1 CD8^+^ T‐cell‐induced tumour ferroptosis

It is well established that CD8^+^ T cells effectively induce tumour cell apoptosis via releasing perforin, granzyme and tumour necrosis factor (TNF), as well as Fas ligand‒Fas interaction.^141^, ^142^ Recently, studies have also shown that IFN‐γ secreted from activated CD8^+^ T cells notably prompts tumour cells' ferroptosis via interaction with surface IFN‐γ receptor on tumour cells.^136^, ^137^, ^138^ Wang et al. demonstrated that IFN‐γ can inhibit the system Xc^−^ expression on tumour cells via enhancing STAT1/IRF1 signalling and inducing the STAT1 abundance on the promoter region of SLC7A11, one subunit of system Xc^−^, thus impairing cystine intake and inducing tumour ferroptosis.^136^ Another study also confirmed the importance of IFN‐γ in hepatocellular carcinoma cell ferroptosis.^143^ Moreover, the combination of IFN‐γ and ferroptosis inducers can cause tumour cell cycle arrest in G0/G1 phase. IFN‐γ derived from CD8^+^ T cells can increase the tumour vulnerability to radiotherapy and immunotherapy by synergistic repressing tumour SLC7A11 expression to promote tumour cell ferroptosis.^138^ The combination of IFN‐γ and arachidonic acid (AA) rewire cancer cell lipid metabolism via ACSL4, further activating PUFA and elevating their susceptibility to ferroptosis in immunotherapy‐relevant settings.^144^, ^145^ IFN‐γ combined with mefloquine‐treated lung cancer cells and melanoma significantly reinforces tumour ferroptosis by activating LPCAT3 expression and sensitising tumour cells to PD‐1 blockade.^146^ Moreover, IFN‐γ combined with iron dextran (FeDx) activates TFR1 expression in lung cancer cells via ferritin autophagy and ferritinophagy, which induces tumour cell ferroptosis and increased T‐cell infiltration in the TME, as well as increasing tumour vulnerability to immunotherapy.^147^ The miRNA profiles were also altered in IFN‐γ‐induced tumour ferroptosis.^148^ The expression of miR‐21‐3p was upregulated in IFN‐γ‐treated melanoma cells, which can promote ROS generation and lipid peroxidation and further aggravate ferroptosis. Furthermore, using a nanoparticle carrier to load and deliver miR‐21‐3p can facilitate the efficacy of the anti‐PD‐1 antibody through reinforcing IFN‐γ‐driven ferroptosis. Recently, Gao et al. have revealed that another type I family member IFN‐κ also drives tumour ferroptosis in combination with AA via the IFNAR/STAT1/ACSL4 axis.^149^ Meanwhile, the chimeric antigen receptor (CAR) T‐cell engineered with IFN‐κ enhances the anti‐tumour efficacy of CAR T therapy. Thus, ferroptosis inducers such as IFN‐γ seem to be promising agents for cancer treatment alone or cooperating with cancer immune checkpoint blockade. To specifically drive tumour ferroptosis, a small‐molecule compound N6F11 was identified that can selectively induce the ubiquitination and degradation of GPX4 in tumour cells but not in immune cells, which can further enhance the function of CD8^+^ T cells and the sensitivity of tumour cells to immunotherapy.^150^ These profound findings have the potential to establish a more safe and effective approach to trigger tumour ferroptosis without impaired anti‐tumour immunity.

In brief, the activated CD8^+^ T‐cell‐derived IFN‐γ and its family member IFN‐κ become powerful ferroptosis inducers to mediate tumour cell ferroptosis. Increasing studies have focused on the efficacy of IFN‐γ in combination with other cancer therapies such as immunotherapy and radiotherapy. Moreover, small‐molecule compounds that directly and selectively induce tumour ferroptosis are currently being developed.

### 6.2 Regulation of CD8^+^ T‐cell ferroptosis

In the development of inflammation and tumours, ferroptosis also plays a crucial role in the exhaustion and viability of CD8^+^ T cells, which disrupts the CD8^+^ T‐cell‐mediated anti‐tumour immunity and anti‐virus immunity. Tian et al. have found that ferroptosis occurs in exhausted CD8^+^ T cells under LCMV infection condition, which triggers the compromised expansion of virus‐specific CD8^+^ T cell.^151^ The ferroptosis of CD8^+^ T cells can be driven by enriched lipids and oxide lipids, such as fatty acids within the TME, via interaction with the surface receptor CD36.^9^, ^140^ Meanwhile, the elevated CD36 expression on the intratumoural CD8^+^ T cells was prompted by cholesterol, which suggests the ferroptosis‐promoting effects of cholesterol metabolism on CD8^+^ T cells.^9^ On the contrary, cholesterol biosynthesis confers ferroptosis resistance of tumour cells.^152^, ^153^ The main reason for this difference may be the intensity of the intracellular ferroptosis defense system. Prostaglandin E2 (PGE2) is an important immune response mediator and is enriched in the TME which also induces CD8^+^ T‐cell ferroptosis.^154^ PGE2 impairs IL‐2 sensing by reducing the IL‐2Rγc chain expression and thus compromising mTOR‐peroxisome proliferator‐activated receptor γ coactivator 1α (PGC1α) signalling, which further causes oxidative stress and ferroptosis in tumour‐infiltrating CD8^+^ T cells.^155^ Thus, CD8^+^ T‐cell ferroptosis can be regulated by various metabolites in the TME, and selectively targeting CD8^+^ T‐cell ferroptosis may represent an effective immunotherapy approach. Interestingly, an integrated bioinformatic database analysis showed that elevated ferroptosis suppressors are positively related to T‐cell exhaustion and cytotoxic CD8^+^ T‐cell evasion in glioblastoma, which further suggests the complexity of ferroptosis in the TME.^156^ Currently, more and more researchers have focused on the intrinsic ferroptosis regulatory mechanisms of CD8^+^ T cells. Ping et al. manifested that intratumoural CD8^+^ T cells with phospholipid phosphatase 1 (PLPP1) deficiency regulated by PD‐1 signalling have more sensitivity to unsaturated fatty acid‐mediated ferroptosis.^157^ Moreover, extracellular cystine is preferentially taken up by tumour cells that highly express SLC7A11 but affect intratumoural CD8^+^ T cells function containing compromised GSH synthesis, CD36‐governed ferroptosis occur and exhausted markers expression.^158^ Furthermore, SLC2A3 also known as a glucose transporter was upregulated in tumour‐infiltrating CD8^+^ T cells and negatively associated with the proliferation and function of CD8^+^ T cells via potentially driving CD8^+^ T‐cell ferroptosis.^159^ However, GCLC overexpression effectively reduces the glutamate accumulation and ferroptosis properties of CD8^+^ T cells, rejuvenating tumour suppressive activity.^158^ The expression of GPX4 is adversely linked with CD8^+^ T‐cell ferroptosis. Interestingly, tumour‐infiltrating CD8^+^ T cells have more abundant GSH and higher lipid ROS levels than their splenic counterpart.^160^ Moreover, the GSH‐GPX4 axis is conducive to inducing the expansion, survival and anti‐tumour response of CD8^+^ T cells, which can be hindered by the adenosine A2A receptor (A2AR). Furthermore, using the A2AR inhibition strategy combined with lipophilic antioxidants such as liproxstatin‐1 can prohibit the ferroptosis of CD8^+^ T cells and sustain the functionality and proliferation of adoptive cell therapy. Enforced GPX4 expression induces effector cytokines secretion and decreases lipid peroxidation accumulation in CD8^+^ T cells.^140^ GPX4 inhibitors prefer to induce ferroptosis of intratumoural CD8^+^ T cells rather than B16 melanoma cells and MC38 colorectal cancer cells.^139^ Ectopic expressed FSP1 and GPX4 promote the resistance of CD8^+^ T cells to ferroptosis in vivo. In contrast, ACSL4 deletion can protect CD8^+^ T cells from ferroptosis but impairs anti‐tumour activity. Furthermore, the supernatant derived from GPX4‐loaded oncolytic vaccinia virus (OVV‐GPX4) can significantly strengthen the function of CD8^+^ T cells in tumour suppression via decreasing the CD36 expression and reducing lipid peroxidation ex vivo, reinforcing the tumour‐killing effects of immunotherapy in Panc02 mouse model.^161^ It suggests GPX4 may be a potential cancer immunotherapy target in the future. On the other hand, Lu et al. have revealed that CD8^+^ Tc9 subset with high IL‐9 secretion exhibits robust anti‐tumour activity and longevity, making it a promising candidate for immunotherapy.^162^, ^163^ IL‐9/STAT3 axis upregulated fatty acid oxidation and mitochondrial activity that impeded the lipid peroxidation overload in Tc9 cells and resistance to tumour or ROS‐induced ferroptosis in the TME.^164^ Li et al. have found that DEP domain‐containing protein (DEPDC) 5, an epilepsy susceptible gene, increases the resistance of CD8^+^ T cells to ferroptosis via reducing mTORC1 activity, and thus enhancing anti‐tumour immunity.^165^ Xiang et al. have demonstrated that PCIF1, an RNA N_6_ 2′‐O‐dimethyladenosine (m^6^A_m_) methyltransferase, can reduce the m^6^A_m_ modification on ferroptosis suppressor genes such as FTH1, SLC3A2 and T‐cell activation marker CD69 in CD8^+^ T cells, which further promotes CD8^+^ T‐cell ferroptosis and enhances tumour growth.^166^ Clinically, the PCIF1 levels in T cells have a negative correlation with immunotherapy efficacy. Therefore, it will be beneficial for generating targeted therapy strategies to elucidate the intrinsic dysfunctional mechanisms and inducements involved in the process of CD8^+^ T‐cell ferroptosis.

Despite the prevalent mutual promotion of ferroptosis between tumour cells and CD8^+^ T cells, the regulators involved in this process exhibited significant differences. The combination of AA and IFN‐γ straightforwardly triggers the tumour ferroptosis mechanism. At the same time, intratumoural CD8^+^ T cells with high ferroptosis vulnerability are driven by the enriched fatty acids and imbalanced amino acids in the TME. Moreover, although ferroptosis‐related agents present exciting potential for cancer therapeutics and immunomodulatory capabilities, there are several critical considerations regarding their safety profiles and potential adverse effects. For instance, the superior susceptivity of intratumoural CD8^+^ T cells to ferroptosis poses significant challenges to the therapeutic effects of ferroptosis‐related agents. Thus, developing appropriate and selective ferroptosis inducers or inhibitors that shield T cells from ferroptosis in the future could be critical strategies for improving the efficacy of cancer immunotherapy. We also concluded the common ferroptosis‐related reagents that are applied in T cells in Table 2, which may inspire further study on T‐cell ferroptosis.

**TABLE 2:** Common ferroptosis‐related reagents applied in T cells.

Ferroptosis‐related reagents	Mechanisms	Application in T cells
**Inhibitors**		
Ferrostatin‐1	Inhibit lipid peroxidation	Abolish the ferroptosis of GPX4‐deficient T cells.^22^ Rescue ferroptosis of activated GPX4‐deficient Tregs and improve its capability to repress CD4^+^ T‐cell expansion.^23^ Increase the frequency and number of Tfh cells and ICOS expression without altering Tfh proliferation or IL‐21 production.^104^ Inhibit lipid ROS production and ferroptosis in Tc9 cells.^164^ Reduce the ferroptosis of CD8^+^ T cells and enhance the function and proliferation of CD8^+^ T cells.^9^, ^139^, ^157^
Liproxstatin	Inhibit lipid peroxidation	Rescue the memory T cells from ferroptosis.^107^ Rescue ferroptosis of activated GPX4‐deficient Tregs.^23^
Vitamin E (α‐tocotrienol)	Inhibit lipid peroxidation	Promote the survival of T cells.^22^ Rescue the memory T cells from ferroptosis.^107^ Rectify aberrant lipid peroxidation in activated GPX4‐deficient Tregs.^23^ Prevent the ferroptosis of Tfh cells.^104^
ΜUFAs	Inhibit lipid peroxidation	Decrease the susceptibility of T cells to lipid peroxidation and ferroptosis.^29^
Deferoxamine	Iron chelation	Abolish the ferroptosis of GPX4‐deficient T cells.^22^ Prevent the ferroptosis of Tfh cells.^104^ Suppress the expression of PFKFB4 in CD4^+^ T cells.^113^ Reduce ROS levels and partially rescue the death of PDK1‐deficient Τregs.^123^ Inhibit the lipid peroxidation in Tc9 cells.^164^ Restrain Th17 cells differentiation.^52^, ^111^ Inhibit clone DNA synthesis of Th1 and Th2 cells.^49^ Promote IFN‐γ production whereas inhibiting IL‐4 secretion.^50^ Enhance IFN‐γ/STAT1 activation in proliferating T cells.^51^
Deferiprone	Iron chelation	Abolish the ferroptosis of GPX4‐deficient Tregs.^23^
Ciclopirox	Iron chelation	Abolish the ferroptosis of GPX4‐deficient Tregs.^23^ Decrease basal and maximal mitochondrial respiration in CD4^+^ T cells.^112^ Reduce Th1 and Th17 differentiation.^52^
Selenium	GPX4 upregulation	Mitigate ferroptosis in T cells, increase Tfh cell numbers and promote antibody responses in infections.^104^
**Inducers**		
Erastin	System Xc^−^ inhibition	Enhance lipid peroxidation and cell death in T and B cells than monocytes or neutrophils.^29^ Rarely impair Treg viability whereas triggering ferroptosis of B16 cells.^23^
Glutamate	System Xc^−^ inhibition	Selectively promote Th1 and impair Th17 differentiation while not affecting Treg.^100^
FIN56	Induce GPX4 degradation	Inhibit the function and proliferation of CD8^+^ T cells.^9^, ^157^ Induce Tc9 cells ferroptosis.^164^
Buthionine sulphoximine	GSH depletion	Decrease ps6 in the activated T effector cells but increase ps6 in Tregs.^114^ Reduce nuclear NFAT and MYC expression in the activated T cells and inhibit T‐cell proliferation.^95^
1S,3R‐RSL3	GPX4 inhibition	Suppress lipid ROS production and ferroptosis in Tc9 cells.^164^ Inhibit the function and proliferation of CD8^+^ T cells.^157^ Reduce CD8^+^ T‐cell ferroptosis and inhibit the cytotoxic cytokine production.^9^
ML162	GPX4 inhibition	Induce high ferroptosis vulnerability of T cells compared with B16 cells.^139^
ML210	GPX4 inhibition	Decrease the PUFA‐PLs abundance in T and B cells.^29^

Abbreviations: GPX4, GSH peroxidase 4; GSH, glutathione; IFN‐γ, interferon‐γ; IL‐4, interleukin‐4; NFAT, nuclear factor of activated T cell; PDK1, 3‐phosphoinositide‐dependent protein kinase 1; PUFA‐PL, polyunsaturated fatty acid‐containing phospholipid; ROS, reactive oxygen species; STAT1, signal transducer and activator of transcription 1; Treg, regulatory T cell.

## 7 CONCLUSIONS AND FUTURE PERSPECTIVE

Ferroptosis‐related mechanisms have been extensively involved in regulating many aspects of T cells, including activation and differentiation, affecting their responsiveness to infection, inflammation and tumour immunity. Above all, we summarised the specific metabolic signals such as mTOR, GSH/GPX4 axis and metabolic byproducts such as PUFAs, intracellular iron, NADPH and ROS in T‐cell biology and ferroptosis, especially from pathological conditions. Besides, we noticed a close cross‐interaction between ferroptosis and autophagy or pyroptosis in T‐cell function, which could be a promising research topic in the future. Due to the metabolic preference among different T‐cell subgroups, including Th17 cells, Tfh cells or Tregs under steady‐state or pathological state, have unique requirements for ferroptotic metabolites such as ROS and GSH, resulting in different susceptibility to ferroptosis. Subsequent studies need to delineate the potential effects of ferroptosis‐related genes on T‐cell development and further clarify the distinct roles of ferroptosis in various T‐cell subgroups. Moreover, current research predominately concentrates on the ferroptosis susceptibility mechanisms and the ferroptosis resistance mediators in T cells from solid tumours, comprehensively analysing the regulatory mode and role of T‐cell ferroptosis between solid tumours and leukaemia represents a potential research direction. The intricate ferroptosis mechanisms resulting from intercellular interactions within the TME pose challenges to applying ferroptosis‐related therapies. Future research should thus aim to delve deeper into the distinct ferroptosis bias of effector T cells and tumour cells, with the ultimate goal of developing corresponding ferroptosis inducers or inhibitors, which could potentially be utilised either independently or in conjunction with other immunotherapies to enhance the therapeutic outcomes for cancer patients. In recent years, the utilisation of nanopolymer delivery systems for targeted delivery of ferroptosis‐related reagents to tumour tissues, coupled with the activation of CD8^+^ T cells and enhancement of tumour ferroptosis, has opened a new direction in cancer treatment. For instance, utilising the graphene oxide and PEI‒PEG material as delivery carriers to load PD‐L1 siRNA and specifically target tumour cells PD‐L1 in that contributing to the CD8^+^ T‐cell‐induced tumour ferroptosis.^167^ The nanoreactor Cu_2−x_Se/ZIF‐8@Era‐PEG‐FA was synthesised to carry the ferroptosis inducer Erastin that can promote tumour ferroptosis but also induce tumour‐associated macrophages polarisation and CD8^+^ T‐cell activation in the triple‐negative breast cancer.^168^ Hence, the integration of nanotechnology with ferroptosis‐related agents offers a novel approach for targeting ferroptosis in T cells during inflammatory processes. This targeted approach not only minimises off‐target effects but also holds promise for developing novel therapeutic interventions that can modulate immune responses and alleviate inflammatory conditions. Remarkably, the combined application of IFN‐γ produced by effector T cells with AA or dextran can significantly induce tumour cell ferroptosis, suggesting the potential roles of IFN‐γ in inducing ferroptosis in autoimmune diseases or infections. Furthermore, the safety and value of ferroptosis inhibitors or inducers in infection, autoimmune diseases, and even anti‐tumour immunity need to be further explored in animal studies and clinical trials.

In summary, the study of ferroptotic T cells represents prospective research with significant implications for understanding and treating various diseases. By continuing to explore the mechanisms and functional consequences of T‐cell ferroptosis and developing novel therapeutic strategies that harness or inhibit this process, we can advance our understanding and select optimal strategies for treating these diseases.

## AUTHOR CONTRIBUTIONS

Xueli Xia wrote the manuscript. Haisheng Wu, Yuxuan Chen and Huiyong Peng discussed and revised the manuscript. Shengjun Wang conceptualised the study and revised the manuscript. All authors read and approved the final manuscript.

## CONFLICT OF INTEREST STATEMENT

The authors declare they have no conflicts of interest.

## ETHICS APPROVAL AND CONSENT TO PARTICIPATE

Not applicable.

## CONSENT FOR PUBLICATION

Not applicable.

## Data Availability

Not applicable.
